# Delayed diagnosis of Netherton syndrome revealed by hair shaft microscopy

**DOI:** 10.1016/j.jdcr.2026.07.038

**Published:** 2026-07-29

**Authors:** Bibek Sharma, Supriya Paudel, Shreya Paudel

**Affiliations:** a Department of Dermatology, Venereology and Leprology, Madan Bhandari Academy of Health Sciences, Hetauda, Nepal; b Department of Biomedical Engineering, College of Biomedical Engineering and Applied Sciences, Kathmandu, Nepal

**Keywords:** atopy, bamboo hair, ichthyosiform erythroderma, Netherton syndrome, trichorrhexis invaginata

## Case description

A 17-year-old girl presented with generalized erythema, scaling, and severe pruritus since early childhood. Symptoms showed intermittent exacerbations, particularly during winter. She also reported recurrent allergic rhinitis and episodic wheezing. There was no family history of similar illness or parental consanguinity.

Physical examination demonstrated annular and polycyclic erythematous scaly plaques with characteristic double-edged scaling over the trunk and extremities, consistent with ichthyosis linearis circumflexa (Fig 1, *A-C*). Scalp examination revealed sparse, short, brittle hair with reduced density and eyebrow thinning (Fig. 2, *A*). Laboratory investigations showed peripheral eosinophilia and markedly elevated serum immunoglobulin E levels. Light microscopy of plucked scalp hair demonstrated ball-and-socket invaginations of the hair shaft (Fig 2, *B*).

**Fig 1:**
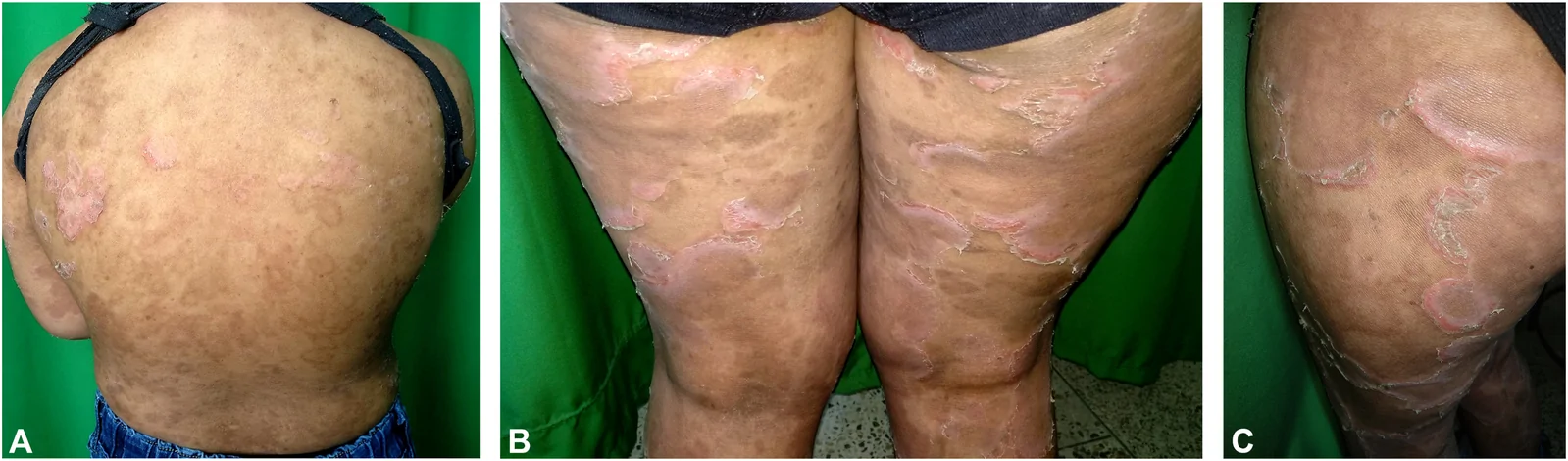
**A-C,** Multiple annular and polycyclic erythematous scaly plaques with characteristic double-edged scaling suggestive of ichthyosis linearis circumflexa over the trunk and extremities.

**Fig 2:**
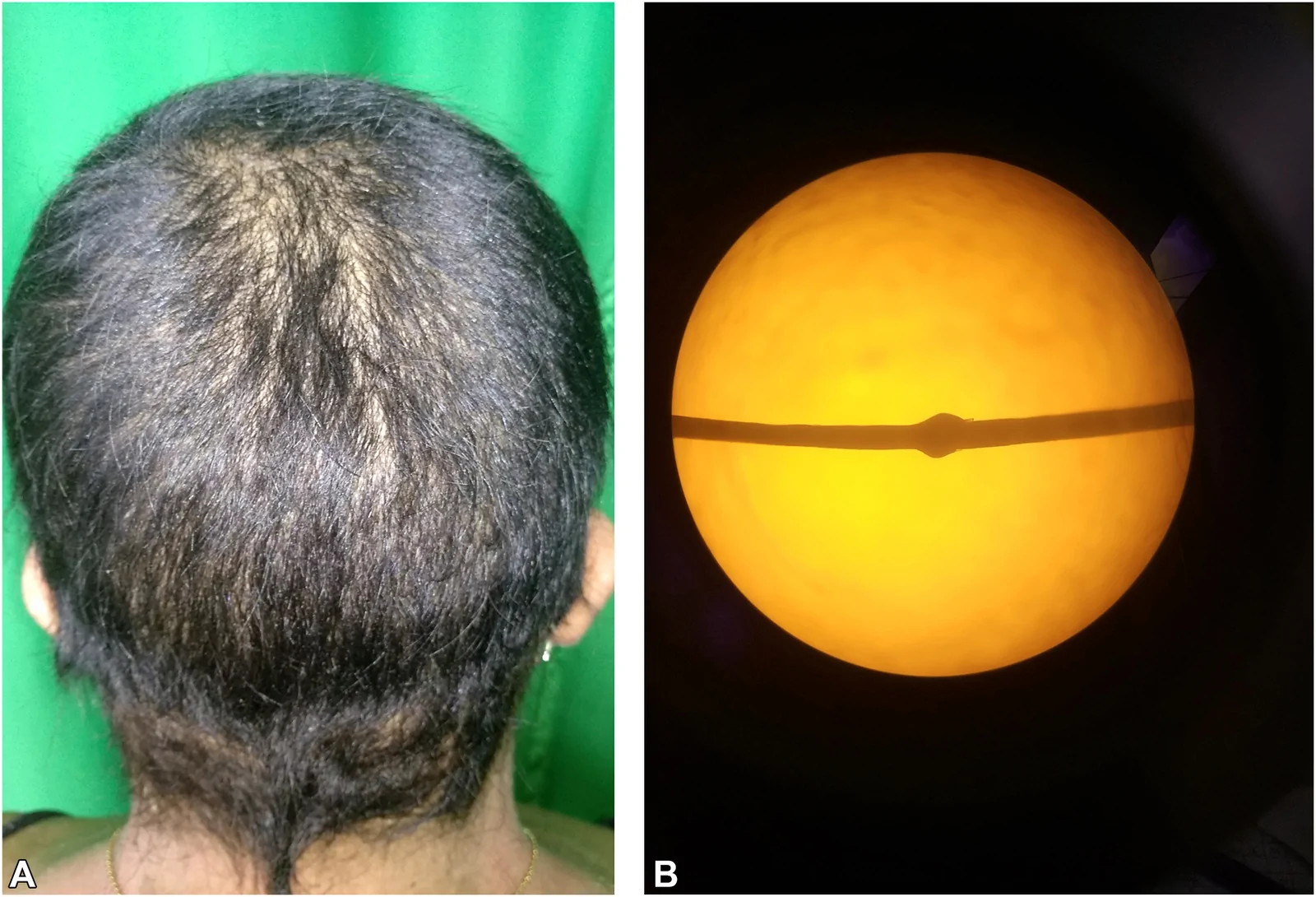
**A,** Sparse, short, and brittle scalp hair with reduced density. **B,** Light microscopic examination of scalp hair showing a characteristic hair shaft abnormality.

**Question 1: Which hair shaft abnormality is characteristically associated with this patient’s condition?**

**A.** Monilethrix

**B.** Pili torti

**C.** Trichorrhexis invaginata

**D.** Trichorrhexis nodosa

**E.** Bubble hair

## Discussion

The correct answer is **C**, trichorrhexis invaginata (“bamboo hair”), the pathognomonic hair shaft abnormality of Netherton syndrome (NS). NS is a rare autosomal recessive genodermatosis caused by mutations in the SPINK5 gene encoding lymphoepithelial Kazal-type-related inhibitor, a serine protease inhibitor that regulates epidermal protease activity and prevents excessive degradation of proteins essential for maintaining the skin barrier. Loss of lymphoepithelial Kazal-type-related inhibitor function results in epidermal barrier dysfunction and atopic diathesis.^1^^,^^2^ The syndrome classically presents with the triad of ichthyosis linearis circumflexa, trichorrhexis invaginata, and atopic manifestations with elevated serum immunoglobulin E levels.

Trichorrhexis invaginata results from telescoping of the distal hair shaft into the proximal portion, producing the characteristic “bamboo hair” appearance.^3^ Although highly suggestive of NS, trichorrhexis invaginata has also rarely been reported in generalized inflammatory peeling skin syndrome. In addition, other hair shaft abnormalities, including trichorrhexis nodosa and pili torti, have also been reported in patients with NS. Hair shaft microscopy is a simple, inexpensive, and valuable diagnostic tool, particularly in resource-limited settings where genetic testing may not be available.

Monilethrix (Option A) is characterized by a beaded appearance of the hair shaft caused by periodic constrictions and is associated with hair fragility and keratosis pilaris. Pili torti (Option B) refers to flattened hair shafts twisted along their longitudinal axis and may occur in disorders such as Menkes disease, Björnstad syndrome, ectodermal dysplasias, and NS. Trichorrhexis nodosa (Option D) is characterized by nodular fractures of the hair shaft, leaving a frayed or split-end appearance. It may be congenital or acquired secondary to physical or chemical trauma and has also been reported in NS. Bubble hair (Option E) results from heat-induced damage to the hair shaft and is characterized by air-filled cavities within the hair.

Although NS typically manifests during infancy, the diagnosis is frequently delayed until later childhood because characteristic hair shaft abnormalities may not be demonstrable early in life owing to sparse hair growth. In addition, the clinical features often overlap with severe atopic dermatitis and congenital ichthyoses. Recognition of characteristic hair shaft findings is essential for accurate diagnosis and appropriate management. Treatment remains largely supportive with emollients, topical anti-inflammatory therapy, and skin barrier protection. Emerging biologic therapies such as dupilumab and secukinumab have demonstrated benefit in refractory disease.^4^^,^^5^

### Declaration of generative AI and AI-assisted technologies in the writing process

None.

## Conflicts of interest

None disclosed.
